# Supplementary material for: Direct penetration of spin-triplet superconductivity into a ferromagnet in Au/SrRuO3/Sr2RuO4 junctions
Source: Nat Commun. 2016 Oct 26;7:13220. doi: 10.1038/ncomms13220 (PMC5095176; doi:10.1038/ncomms13220)
Supplement: Supplementary Information — Supplementary Figures 1-8, Supplementary Table 1, Supplementary Notes 1-5 and Supplementary References. [file ncomms13220-s1.pdf]

Supplementary figures

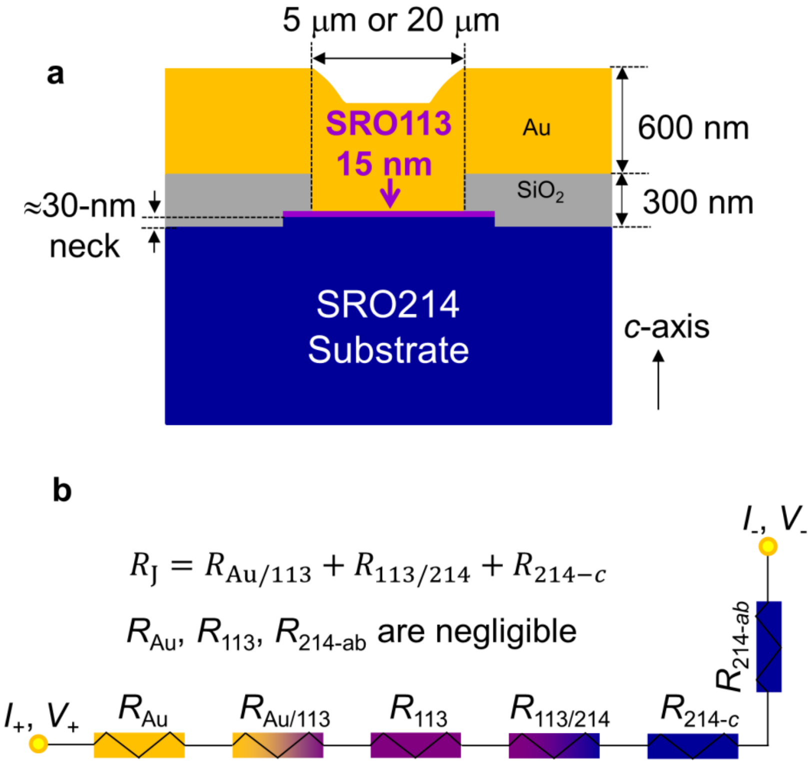

Supplementary Figure 1. A, Schematic of a Au/SRO113/SRO214 junction. A 15-nm thick SRO113 layer was etched along with ≈30-nm thick SRO214 substrate layer. To isolate the top Au electrodes from SRO214 superconductor, a 300-nm thick SiO_2 layer was sputtered. This layer also covers the sides of the SRO113 pad. **b,** A series resistance model of the junction.

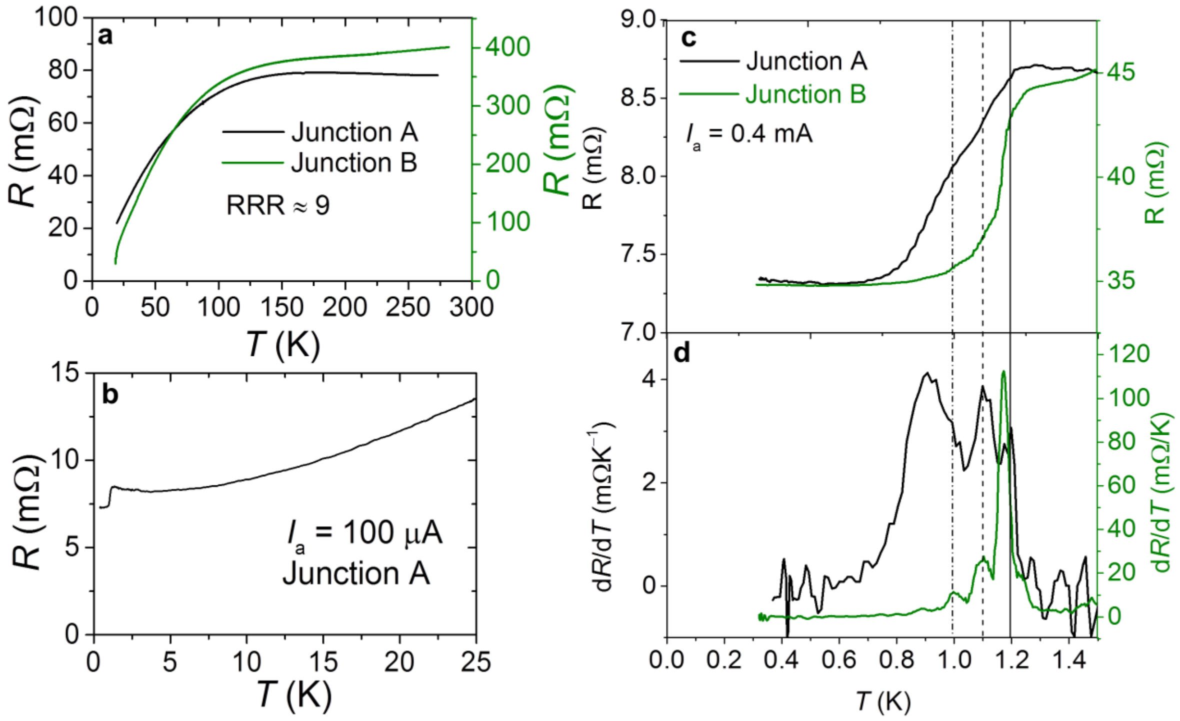

Supplementary Figure 2. a, Temperature dependent resistance $R(T)$ at higher temperatures (250 K to 20 K) of junction A (black curve) and B (green curve). **B,** $R(T)$ of junction A at lower temperatures (25 K to 0.3 K). Superconducting transition is observed at 1.22 K with lowest resistance ≈7.2 Ω. **c,** Comparison of the resistance behavior near the transition of both junctions A and B. **d,** Temperature derivative of the resistance data shown in (**c**). Three clear peaks are observed, corresponding to three transitions.

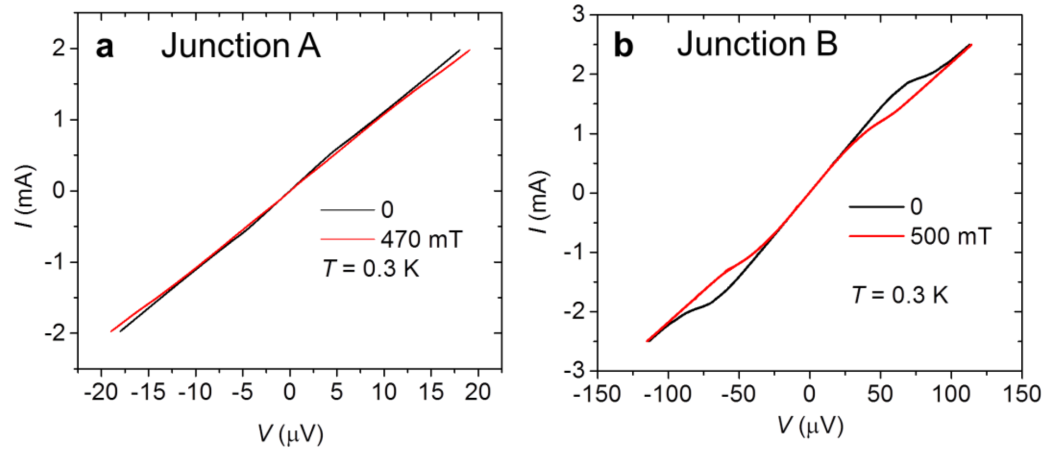

Supplementary Figure 3. a, Current-Voltage (*I*-*V*) curves of junction A measured at 0.3 K in magnetic field of 0 mT (black curve) and 470 mT (red curve) applied along the *ab* plane. **b,** *I*-*V* curves of junction B at 0 mT (black) and 500 mT (red) measured at 0.3 K.

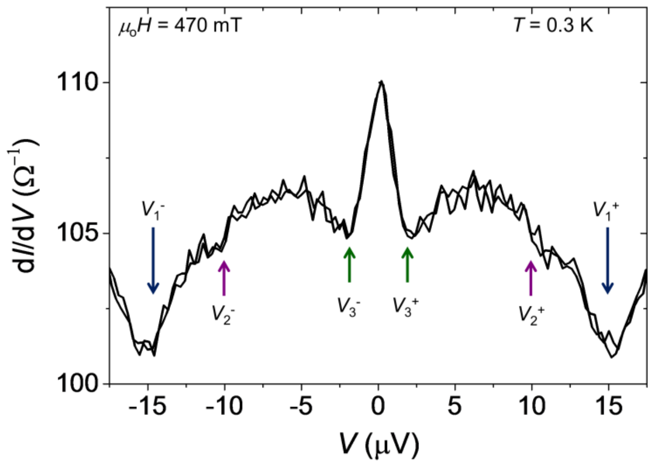

Supplementary Figure 4. Example of the evaluated characteristic voltages for the differential conductance d*I*/d*V* as a function of the bias voltage of junction A at 0.3 K and 470 mT. The characteristic voltage are indicated with vertical arrows.

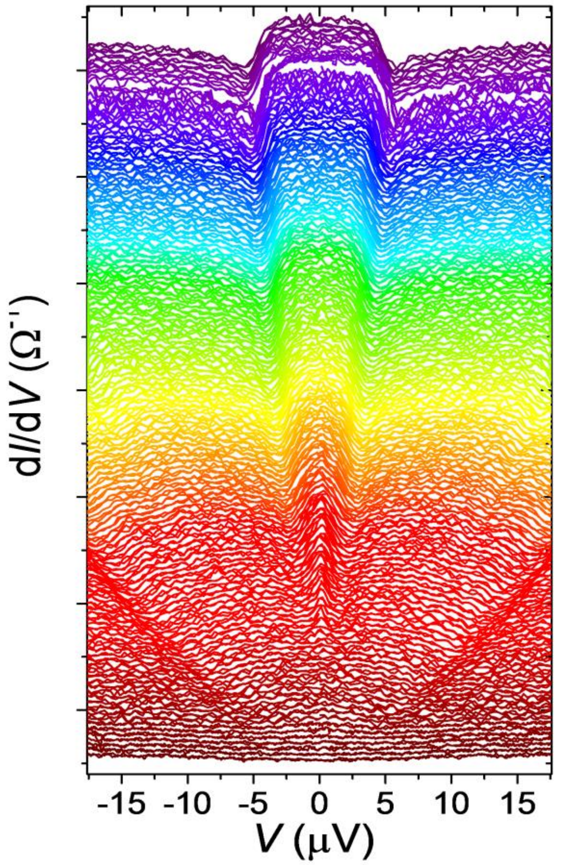

Supplementary Figure 5. Differential conductance as a function applied field along the ab-plane at 0.3 K of junction

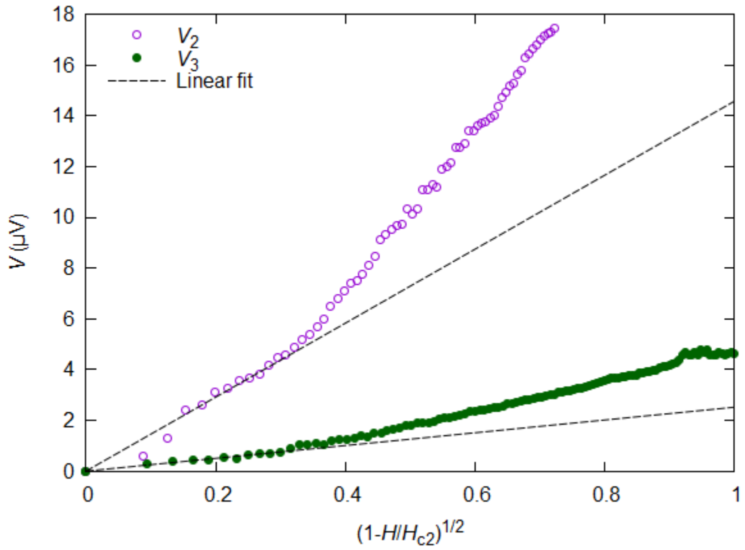

Supplementary Figure 6. Voltage V_2 and V_3 at 0.3 K as a function $(1-H/H_{c2})^{1/2}$ with linear fit. It shows that both V_2 and V_3 are following square root behavior close to the transition, however at lower $(1-H/H_{c2})$ is also

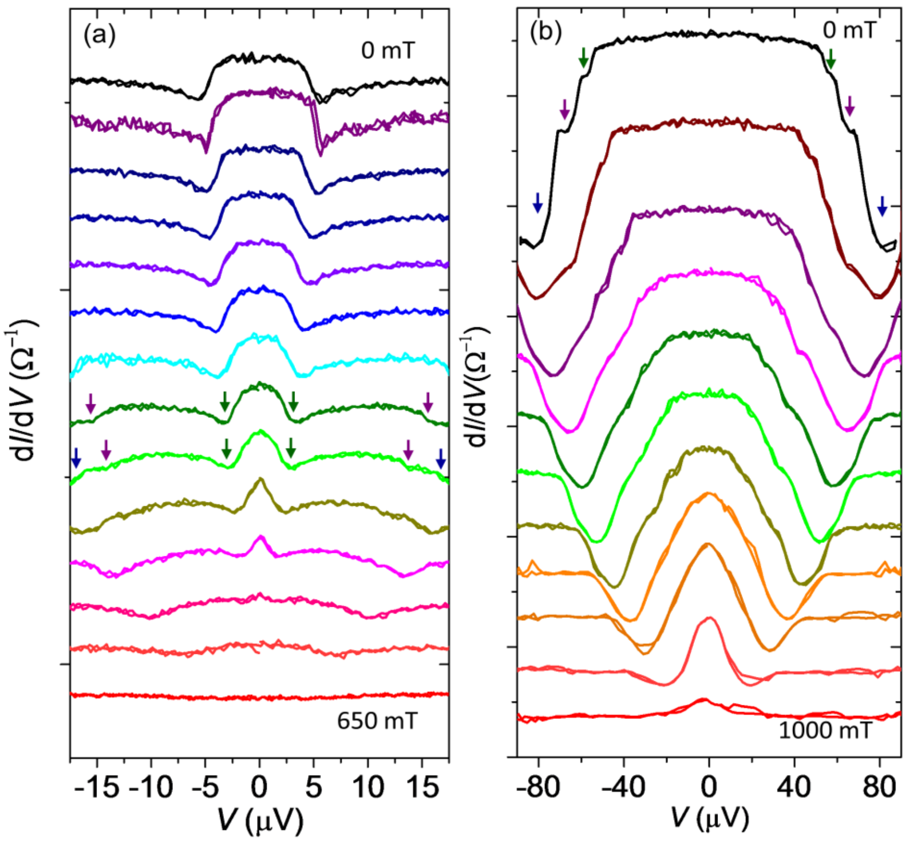

Supplementary Figure 7. a, d*I*/d*V* vs bias voltage of junction A at 0.3 K measured at various applied fields (in-plane) with field interval of 50 mT. **b,** d*I*/d*V* of junction B at 0.3 K with field interval of 100 mT. Three transitions are clear for both junctions A and B as indicated with arrows.

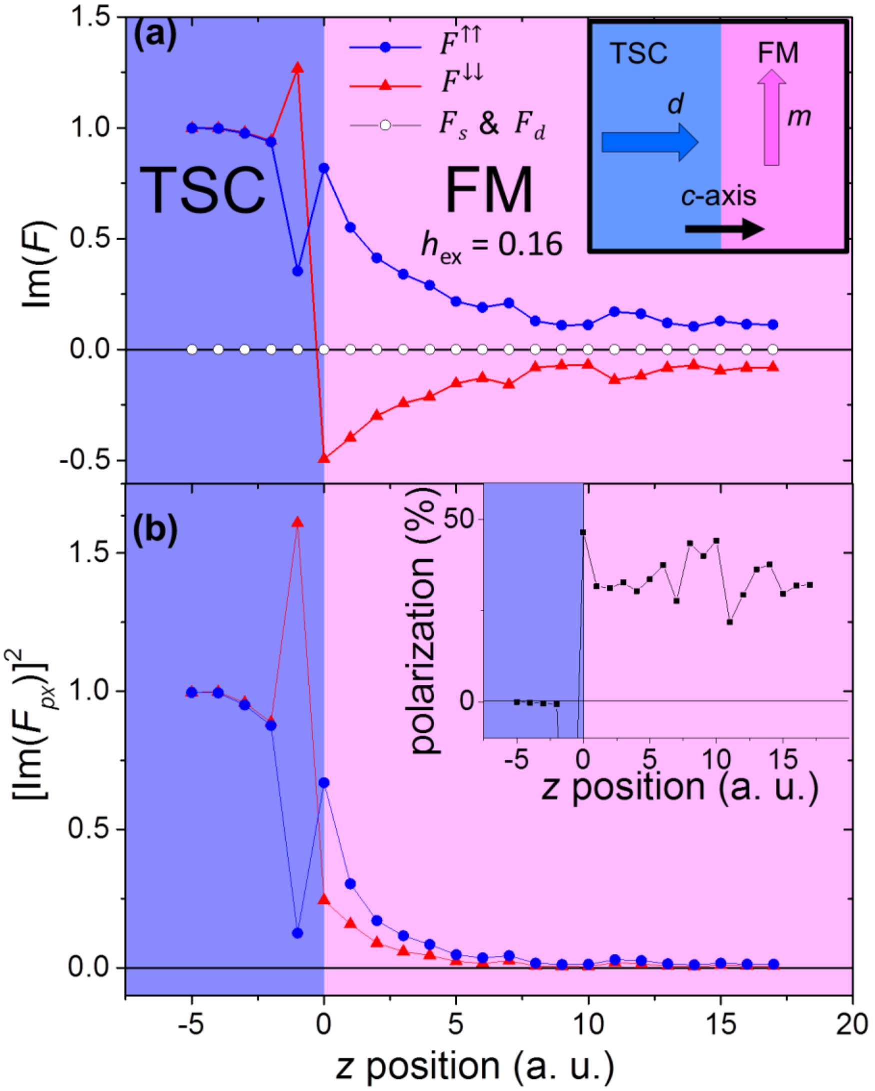

Supplementary Figure 8. a, Calculated spatial variation of the imaginary part of the normalized p_x-wave pair correlation F for spin ↑↑ (blue closed circles) and ↓↓ (red closed triangles) configurations with the spin quantization axis along the x axis. The exchange field h_{ex} is assumed to be $0.16t$, where t is the hopping amplitude. The inset shows a schematic of the model junction. In this configuration of the FM/TSC junction, F_{px} and F_{py} are equal and spin singlet s-wave and d-wave correlations are zero (open circles). **b,** Spatial variations of the square of $\mathrm{Im}(F_{px})$ for both Cooper-pair spin directions. The inset shows the polarization $\tilde{P}$ deduced from $[\mathrm{Im}(F_{px})]^2$.

Supplementary Table 1. Comparison of various parameters of junction A and B. Characteristic voltages are measured at 500 mT. Note that $R_{\mathrm{N}} = R_{\mathrm{J}} - R_{214-\mathrm{neck}}$ and A_s is the area between Au and SRO113. Junction length of both junctions is 15-nm.

	Junction Area b/w SRO113/SRO214 (μm^2)	Junction Area b/w Au/SRO113 (μm^2)	V_1 (μV)	V_2 (μV)	V_3 (μV)	R_{N} (mΩ)	$R_{\mathrm{N}}A_{\mathrm{S\text{-}Au/113}}$ (10^{-12} Ωm^2)	ξ^*_{113} (nm)
Junction A	25 × 25	20 × 20	14.62	10.16	1.77	8.25	3.3	9
Junction B	10 × 10	5 × 5	52.81	32.25	21.20	37.5	0.94	35

Supplementary Note 1.

Considering the structure of the junction (Supplementary Figure 1a), we construct the series resistance model for the overall measured resistance, as shown in Supplementary Figure 1b. The junction resistance consists of the resistance of the Au electrode R_{Au}, the Au/SRO113 interface resistance $R_{\mathrm{Au/113}}$, the resistance of the SRO113 layer R_{113}, the SRO113/SRO214 interface resistance $R_{113/214}$, the resistance of SRO214 in the neck part $R_{\mathrm{214\text{-}neck}}$, and the resistance of the bulk SRO214 R_{214}. Since two electrodes are connected on the side (*ac*-plane) of SRO214 bulk, therefore in-plane resistance is dominating for the bulk substrate. Figure S2 shows the resistance curves below 250 K. From known resistivity values of Au (≈36 μΩ at 4 K), SRO113 (≈5 μΩ at 4 K) and SRO214 (≈20 μΩ at 4 K: along the *ab*-plane)[1-3] R_{Au}, R_{113} and R_{214} are negligibly small for all investigated temperature range. However, $R_{\mathrm{214\text{-}neck}}$ of junction A is estimated to be 8 mΩ at 250 K and 0.5 mΩ at 2 K using the values ρ_c (250 K) ≈ 16 mΩcm and ρ_{co} (2 K) ≈ 1 mΩcm.[3] Thus, $R_{\mathrm{214\text{-}neck}}$ contribution is about 10% of the total resistance both at 250 K and 2 K. The rest of the resistance arises at the interfaces. This non-negligible contribution of $R_{\mathrm{214\text{-}neck}}$ is also supported by the fact that the $R(T)$ curve of junction A shown in S2a is similar to the $\rho_c(T)$ curve of SRO214[3]. In addition, resistance drop between first and second transition $\Delta R \approx$ 0.45 mΩ almost matches with the estimated $R_{\mathrm{214\text{-}neck}}$ at 2 K. This fact, supporting again our series resistance model, indicates that the first superconducting transition originates at the SRO214 neck, as discussed in the main text. Similarly, $R_{\mathrm{214\text{-}neck}}$ for junction B is estimated to be 50 mΩ at 250 K and 3 mΩ at 2 K. The latter is compared with first resistance drop of 4 mΩ. Thus similar conclusion is deduced for junction B. The interpretation that the first superconducting transition originates from the neck part is supported by the critical current estimation as explained in the main text.

Based on this analysis, we evaluate the resistance of the interfaces $R_{\mathrm{int}} = R_{\mathrm{Au/113}} + R_{113/214}$ as R_{int} (2 K) ≈ 8.25 mΩ and R_{int} (250 K) ≈ 67 mΩ for junction A and R_{int} (2 K) ≈ 37.5 mΩ and R_{int} (250 K) ≈ 350 mΩ for junction B. For both junctions, R_{int} is substantially reduced by decreasing temperature. This metallic behavior is consistent with highly

conducting interface[4], providing basis for our interpretation of the bias voltage dependent conductance data in terms of the Andreev reflection[5].

Supplementary Figure 2d shows the temperature derivative of resistance. It exhibits three main peaks corresponding to the resistance variations. Interestingly, the peak-top temperatures for the first two peaks are similar for both junctions A and B. These observations suggest that first two transitions are corresponding to the bulk SRO214-neck and SRO113/SRO214 interface respectively. The third peak is interpreted to arise at the Au/SRO113 interface. Note that the shape of this peak varies depending on junctions. Junction A exhibits a broader peak, whereas junction B has a rather sharp peak. Also, the third peak appears at a lower temperature for junction A compared with junction B. These facts indicate that the induction of superconducting correlations in SRO113 layer for junction A is weaker than to junction B owing to the different interface transparencies.

Supplementary Note 2.

We mainly measure the current-voltage (*I*-*V*) curves and take the derivative to analyze the data. Supplementary Figure 3 presents raw *I*-*V* curves at zero and finite magnetic fields. From these *I*-*V* curves, the critical current corresponding V_1 (discussed in the main text) for junction A (junction B) at around 0.5 T is 1.6 mA (1.2 mA), which yields the critical current density at the neck part to be $\approx 1.2 \times 10^7$ A/m^2 ($\approx 2.5 \times 10^7$ A/m^2). These critical current density values for both of these junctions are of the same order. This fact supports our argument that V_1 arises from the critical current density of the bulk SRO214 at the neck part.

After taking *I*-*V* curves and calculate the derivative d*I*/d*V* as a function of the bias voltage, the characteristic voltages V_1, V_2 and V_3 are evaluated by taking the average of positive and negative values of voltages: e.g. $V_1 = 1/2(V_1^+ + V_1^-)$ (see Supplementary Fig. 4). We chose this approach because the observed d*I*/d*V*(*V*) curves are almost symmetric with respect to the sign inversion $V \rightarrow -V$ except for a small offset voltage originating from the thermoelectric effect among metals used for current leads in the cryostat. This offset can be eliminated by the present analysis method.

We also measured the characteristic voltage $V_1 – V_3$ as a function of applied field along the *ab*-plane (Supplementary Fig. 5). To evaluate the $V_2(H)$ and $V_3(H)$ data, we apply the theoretical fit of superconducting gap suppression with applied field, $\Delta(H) = \Delta(0)\sqrt{1-\frac{H}{Hc}}$. It obviously shows that at higher field (close to the transition), V_2 and V_3 are following the square root behavior. But at lower fields the linear behavior is also contributing. Since, V_1 originates from critical current transition therefore we apply the fit only for V_2 and V_3. However, V_1 may also follow the same behavior at higher fields.

Supplementary Note 3: Other possible origins of V_2 and V_3

In the main text, we discuss that the origins of V_2 and V_3 are the Andreev reflection at the SRO113/SRO214 and Au/SRO113 interfaces, respectively. Here, we discuss other possible origins of these multiple energy scales.

(a) Multi-band superconductivity of SRO214

The first possibility is the multi-band superconductivity of SRO214[6]. This oxide has three Fermi surfaces labeled as α, β, and γ. Theoretical calculations[7] and specific heat measurements[8] reveal that the superconducting gap on the γ surface is about 3 times larger than those on the α and β surfaces. This multi-gap nature may induce multiple features in the d*I*/d*V* data. For example, d*I*/d*V* curves in in-plane tunnel junctions exhibit multiple gap-like features whose voltage ratio exactly matches the gap ratio (3.3)[9]. However, in our junctions, the two junctions exhibit the different ratio between V_2 and V_3 (V_2/V_3 = 5.7 for junction A and 1.5 for junction B at 0.5 T) in both junctions, V_2/V_3 differs from the gap ratio. In addition, the V_2 and V_3 features persist up to 500 mT, whereas the gaps on the α and β surfaces are believed to be closed at around 150 mT[10] even for $\mu_0 H||ab$-plane. These facts indicate that V_2 and V_3 are related to the interface transparency, but not to the multiple bulk superconducting gaps.

(b) Reduced and induced gaps

The second possibility is that the features of V_2 and V_3 both originates from the SRO113/SRO214 interface. Indeed, in simple SN junctions, multiple gap like features have

been observed[11] and attributed to the reduced superconducting gap close to the interface Δ_{red} in the S side and the induced mini-gap Δ_{ind} in the N side. In this scenario, V_2 corresponds to Δ_{red} and V_3 corresponds to Δ_{ind}. It is theoretically expected that Δ_{red} improves with the reduction of the transparency of the interface. However, in our junctions, V_2 is larger for junction B, which has higher transparency. Thus, this second scenario cannot explain the observed behavior either.

(c) Andreev bound state

The third possibility is that the conductance peak within $\pm V_3$ originates from the enhancement of density of states near the interface due to the formation of the Andreev bound state (ABS)[9], which originates from the *p*-wave superconducting order parameter of SRO214. In this scenario, it is assumed that a tunneling barrier is accidently formed at the SRO113/SRO214 interface. However, for the quasi-two-dimensional *p*-wave state, ABS is not expected for out-of-plane tunnel junctions[9]. If in-plane tunneling occurs through atomic steps at SRO214 substrate surface, a broad hump-like behavior within the bulk superconducting gap should be observed[9]. In addition, the observed flat-top peak shape is less common for tunneling junctions but agrees with Andreev reflection behavior. Therefore, the peak within $\pm V_3$ is not attributable to the tunneling spectrum with the ABS.

Supplementary Note 4.

We summarize important parameters of junction A and B in Supplementary Table 1 to compare. The junction areas are different but the junction length is the same (15-nm thick SRO113 layer). Normal-state interface resistance is defined as $R_{\mathrm{N}} = R_{\mathrm{J}} - R_{214\mathrm{-neck}}$ and surface area A_s is taken between Au and SRO113. The ratio between the junction impedance $Z=R_N A_S$ of junctions A and B is about 3.5. This indicates that the interface transparency of junction B is larger than that of junction A. According to the BTK theory for the Andreev reflection[5], it is expected that the conductance enhancement near $V \sim 0$ should be stronger for junction B with smaller Z. Indeed, dI/dV of junction B is 29.2 Ω^{-1} at $V \sim 0$, which is 49% higher than the conductance at the normal state (dI/d$V \sim 19.6\ \Omega^{-1}$). This enhancement is certainly higher than that for junction A (20% enhancement).

At 0.3 K and 500 mT, junction B has three times higher V_1 and V_2 than junction A. But V_3 of junction B is about 12 times higher than that of junction A. As a result, ξ^*_{113} is enhanced to 35 nm in junction B. This enhancement also agrees with the higher transparency of junction B. Most importantly, our devices exhibit rather high reproducibility.

Supplementary Figure 4 presents the deferential conductance as a function of the bias voltage measured at various applied fields along the ab-plane. At zero field, both junctions exhibits flat-top enhancement of conductance around $V = 0$, characteristics for the Andreev reflection. Three characteristic features V_1, V_2, and V_3 are evident for both junctions. Supplementary Figure 5 shows a complete set of dI/dV data that is used to produce the color map given in the main text.

Supplementary Note 5: Theoretical model

As we explain in the main text, the observed anomaly in the conductance of the SRO113/SRO214 junctions indicates direct penetration of spin-triplet superconductivity into SRO113. To strengthen our interpretations, we performed a theoretical model calculation.

For the calculation, we followed the model described in Ref. 12. We calculated the spatial profile of the spin-polarized (↑↑ and ↓↓) Cooper pair amplitude F for a c-axis oriented FM/TSC junction using a self-consistent Bogoliubov-de Gennes approach on a three-dimensional lattice (solved layer-by-layer). To model the junction, we considered a uniform FM layer with the exchange field corresponding to that of SRO113 $h_{\mathrm{ex}} = 0.16t$ (t is the hopping amplitude) attached onto an ab-surface of a uniform quasi-two-dimensional TSC that exhibits chiral p-wave orbital symmetry $p_x + ip_y$ as Sr_2RuO_4 (see the inset of Supplementary Fig. 8a). The orbital angular momentum L and d-vector describing the superconductivity in the TSC are both assumed to be perpendicular to the interface (*i.e.* along the c axis). We fixed the orientation of the magnetization of the FM layer parallel to the interface (*i.e* along the a axis). The interface is assumed to be uniform and free of

magnetic inhomogeneity. Note that the lattice spacing of our model does not directly correspond to the actual crystal lattices of SRO113 and SRO214.

Supplementary Figure 8a presents the imaginary part of the pair amplitude F with the orbital symmetry of p_x and/or p_y. We calculate $F_{px}^{\uparrow\uparrow}$ and $F_{px}^{\downarrow\downarrow}$ with the quantization axis along the x axis (parallel to the interface: along the magnetization direction). These two components exhibit exponential decay with weak spatial oscillations in the FM layer. Notice that the order parameter of the bulk SRO214 with the quantization axis along the x axis is imaginary if we express the order parameter of SRO214 as a real d-vector (see the relations $\hat{\boldsymbol{z}} = |S_z = 0\rangle = \frac{1}{\sqrt{2}}(|\uparrow\downarrow\rangle_z + |\downarrow\uparrow\rangle_z) = \frac{i}{\sqrt{2}}(|\uparrow\uparrow\rangle_x + |\downarrow\downarrow\rangle_x)$). Thus, we anticipate that imaginary part dominates in the FM layer as well. The anomalous data point ($z = -1$) in the vicinity of the interface arises due to the boundary conditions at the interface. The square of F is proportional to the Cooper pair density, which is shown in Supplementary Fig. 8b. We found that the spin polarization deduced from the imaginary part of F, $\tilde{P} = \frac{\mathrm{Im}(F\uparrow\uparrow)^2 - \mathrm{Im}(F\downarrow\downarrow)^2}{\mathrm{Im}(F\uparrow\uparrow)^2 + \mathrm{Im}(F\downarrow\downarrow)^2}$, is about 30% inside the FM-layer. This value agrees with the experimental value of the ferromagnetic spin polarization of SRO113[13]. Note that $\tilde{P}$ is almost constant inside the FM. Interestingly, it is revealed that $F_{px} = F_{py}$ for the present configuration where the spins of Cooper pairs and magnetization are aligned. Thus, by taking an imaginary linear combination of p_x and p_y, a chiral-p-wave correlation can arise in the FM layer. We also performed calculations of spin-singlet pair amplitudes with s-wave and d-wave symmetries and clarified that these correlations cannot emerge at a smooth FM/TSC interface.

Because the inversion symmetry breaks at the interface, the odd-frequency s-wave spin-triplet correlation can be generated at the FM/TSC interface as well. In case of a clean system with a smooth interface, the amplitude of such correlation is very small compared to that of the directly penetrating p-wave correlation. The detailed model calculations considering such odd frequency pairs as well as variation of parameters will be discussed in a separate publication.

In summary, our calculations reveal that a direct penetration of *p*-wave spin-triplet correlation into a FM out of a TSC is possible at a *c*-axis oriented FM/TSC interface that is smooth, uniform, and free of magnetic inhomogeneity.

Supplementary References

1. Gupta, A. K., Cretinon, L., Moussy, N., Pannetier, B., & Courtois, H., Anomalous density of states in a metallic film in proximity with a superconductor. *Phys. Rev. B* **69,** 104514 (2004).
2. Koster, G., et al., Structure, physical properties, and applications of $SrRuO_3$ thin films. *Rev. Mod. Phys.* **84,** 253 – 298 (2012).
3. Hussey, N. E., Mackenzie, A. P., Cooper, J. R., Maeno, Y., Nishizaki, S. and Fujita, T., Normal-state magnetoresistance of Sr_2RuO_4. *Phys. Rev. B* **57,** 5505 (1998).
4. Anwar, M. S., et al., Ferromagnetic $SrRuO_3$ thin-film deposition on a spin-triplet superconductor Sr_2RuO_4 with a highly conducting interface. *Appl. Phys. Express* **8,** 015502 (2015).
5. Blonder, G. E., Tinkham, M., & Klapwijk, T. M., Transition from metallic to tunneling regimes in superconducting microconstrictions: Excess current, charge imbalance, and supercurrent conversion. *Phys. Rev. B.* **25,** 4515 (1982).
6. Mackenzie, A. P., & Maeno, Y., The superconductivity of Sr_2RuO_4 and the physics of spin-triplet pairing. *Rev. Mod. Phys.* **75,** 657–712 (2003).
7. Nomura, T., & Yamada, K., Detailed investigation of gap structure and specific heat in the *p*-wave superconductor Sr_2RuO_4. *J. Phys. Soc. Jpn.* **71,** 404– 407 (2002).
8. NishiZaki, S., Maeno, Y., & Mao, Z., J. *Phys. Soc. Jpn.* **69,** 572–578 (2000).
9. Kashiwaya, S., et al. Edge states of Sr_2RuO_4 detected by in-plane tunneling spectroscopy. *Phys. Rev. Lett.* **107,** 077003 (2011).
10. Deguchi, K., Mao, Z. Q., & Maeno, Y., Determination of the superconducting gap structure in all bands of the spin-triplet superconductor Sr_2RuO_4. *J. Phys. Soc. Jpn.* **73,** 1313–1321 (2004).
11. Sueur, H. L., Joyez, P., Pothier, H., Urbina, C., & Esteve, D., Phase controlled

superconducting proximity effect probed by tunneling spectroscopy. *Phys. Rev. Lett.* **100,** 197002 (2008).

12. Terrade, D., Gentile, P., Cuoco, M., & Manske, D., Proximity effects in spin-triplet superconductor-ferromagnet heterostructure with spin-active interface. *Phys. Rev. B* **88**, 054516 (2013).

13. Koster, G. *et al.* Structure, physical properties, and applications of $SrRuO_3$ thin films. *Rev. Mod. Phys.* **84,** 253 – 298 (2012).
